# Parathyroid carcinoma presenting with chronic renal failure and single pulmonary metastasis: A case report

**DOI:** 10.1016/j.ijscr.2019.10.086

**Published:** 2019-11-06

**Authors:** Lucia Stella Curto, Rita Gervasi, Francesca Caracciolo, Nadia Innaro

**Affiliations:** a Unit of Clinical Surgery, Department of Medical and Surgical Sciences, Magna Graecia University Medical School, Catanzaro, Italy; b Unit of Endocrine Surgery, A.O.U. Mater Domini, Catanzaro, Italy

**Keywords:** Parathyroid carcinoma, Pulmonary metastasis, Hypercalcemia, Chronic renal failure

## Abstract

**Introduction:**

Parathyroid carcinoma is a rare malignancy. Our case report is unusual for its presentation. The patient referred symptoms compatible with CRF; nodular lesions to parathyroid glands and an elevated PTH induced physician to hypothesize secondary hyperparathyroidism. The presence of haemoptysis gave us suspicion for malignant neoplasm but only histological examination allowed us to recognize the nature of the lesion, because even PET-CT didn’t detect, an hypercaptation in the parathyroids.

**Presentation of the case:**

The case concerns a 59 years old female with the following symptoms: asthenia, oliguresis, nausea, haemoptysis and very high levels of calcium and PTH. Laboratory findings (PTH 570 pg/ml; Ca 12.20 mg/dl; P 1.8 mg/dl), ultrasound, CT scan and parathyroid scintigraphy, associated with clinical data, indicated a parathyroid carcinoma with single pulmonary metastasis of the upper lobe of the left lung, confirmed by histological examination and immunochemistry. Cytology performed with bronchoscopy was negative. Surgery (“en bloc” parathyroidectomy on adjacent structures, lymph node dissection near recurrent nerve and atypical lung resection) was effective, with normalization of calcium and PTH levels and disappearance of symptoms after 48 h. After six months, no signs of local recurrence or metastases were observed.

**Discussion:**

No clinical or bio-humoral data allows a preoperative diagnosis of parathyroid carcinoma. Only with definitive pathology and immunochemistry it is possible to differentiate an adenoma from a carcinoma.

**Conclusion:**

In patients with CRF it’s difficult to diagnose parathyroid carcinoma, because its presentation mimics the most common secondary hyperparathyroidism; the concomitant presence of metastases should lead us to suspect malignant parathyroid lesions.

Surgery is the only effective therapy and therefore should be always performed.

## Highlights

• In patients with CRF it’s difficult to diagnose parathyroid carcinoma.
• The concomitant presence of metastases should lead us to suspect malignant parathyroid lesions.
• In literature there are no cases of parathyroid carcinoma in patients with CRF (chronic renal failure) diagnosed by means of pulmonary metastasis.

## 1 Introduction

Parathyroid carcinoma is a rare endocrine malignancy, difficult to define clinically and histopathologically. It represents less than 0.005 % of all cancer [1]. Less than 1 % of sporadic primary hyperparathyroidism (PHPT) is identified as carcinoma, even if in Japanese population there is an incidence of 5 % [2,3].

Generally, parathyroid carcinoma appears with hypercalcemia symptoms, rarely with symptoms of compression/invasion or neck mass. Histological diagnosis, without metastases or clear signs of invasion in the surrounding tissues, is not simple [4].

We report a singular case of parathyroid carcinoma with single pulmonary metastasis.

This work has been reported in line with the SCARE criteria [5].

## 2 Case report

**Fig. 1:**
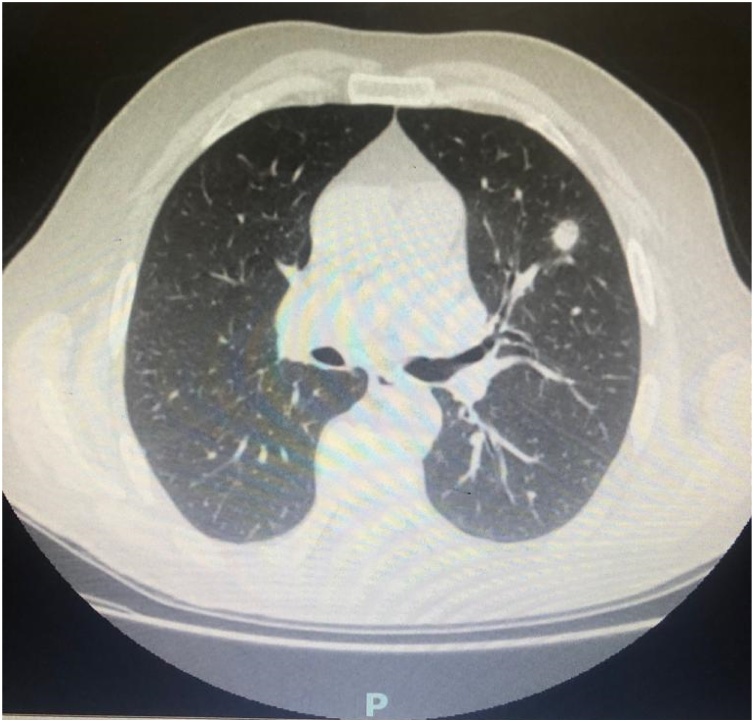
CT imagine of pulmonary metastasis.

A 59-year-old woman was admitted to the hospital for asthenia, nausea, oliguresis and haemoptysis. In her medical history, in 1978 she underwent kidney transplant for polycystic kidney disease; despite this treatment she had IV stage KDOQI chronic kidney disease in hemodialysis treatment; she suffered from anemia, diffuse skin xerosis, multiple warts, osteodystrophy with osteonecrosis and osteoporosis. CT of the Chest revealed to the left upper pulmonary lobe a 10 mm nodular formation, with significant enhancement compatible with neoplastic lesion [Fig. 1]. There was also a nodular lesion localized in the left lobe of thyroid identified as a parathyroid adenoma. PET–CT detected hypercaptation only in the pulmonary formation. Cytology performed with bronchoscopy was negative.

**Fig. 2:**
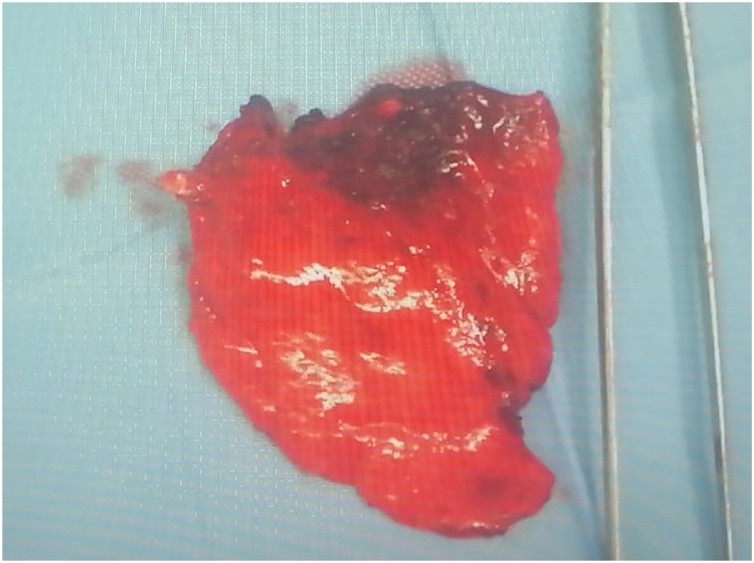
Pulmonary metastasis.

Laboratory data were as follows: PTH 570 pg/ml; Ca 12.20 mg/dl; P 1.8 mg/dl; Hb 10,6 g/dl; HCT 31,5 %; creatinine 4,16 mg/dl;

The patient underwent atypical lung resection; pathological examination was compatible with metastatic lesion from parathyroid carcinoma [Fig. 2].

**Fig. 3:**
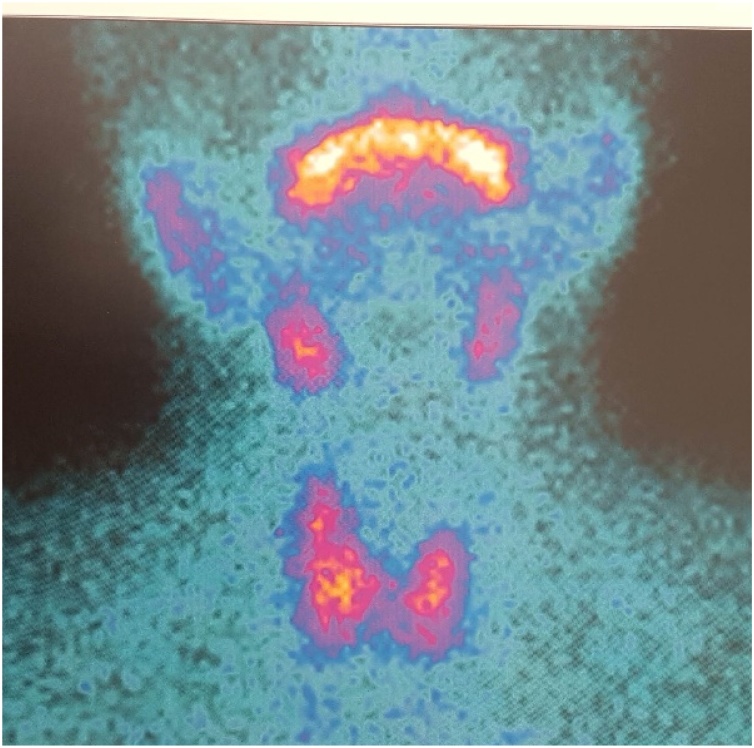
Parathyroid scintigraphy.

After about a month, the patient underwent parathyroid scintigraphy that showed pathological hypercaptation of radionuclide in the lower pole of the thyroid left lobe and in paratracheal region [Fig. 3]. Milder hypercaptation was present on the lower right lobe of the thyroid gland.

Laboratory data were as follows: PTH 1544.5 pg/ml; Ca 14 mg/dl; P 1.4 mg/dl; Hb 10,4 g/dl, creatinine 4,56 mg/dl.

The patient underwent left “en bloc” parathyroidectomy with adjacent structures and lymph nodes dissection near the left recurrent nerve.

Parathyroids appeared almost white, hard and increasing in size. The post-operative period was uneventful and the patient was discharged within 72 h after surgery. Pathological examination of parathyroid glands revealed neoplastic proliferation, capsule invasion and infiltration of muscles, adipose tissue and perithyroidal tissue; thyroid gland was not involved.

On immunohistochemical examination, the tumor cells were positive for TTF1, Cyclin D1 and P16. These results were consistent with parathyroid carcinoma.

After six months, no signs of local recurrence or metastases were observed.

## 3 Discussion

No clinical or bio-humoral data allows a preoperative diagnosis of parathyroid carcinoma. Only with definitive pathology and immunochemistry it is possible to differentiate an adenoma from a carcinoma. Surgery is the only effective therapy and therefore should be always performed. This neoplasm usually relapses, locally first and later with distant metastases. Therefore, after surgery, the patients should always undergo a rigorous follow up program including evaluation of PTH and serum calcium.

The literature suggests that the most important factor that influences the prognosis is the complete removal of the neoplasm. In patients undergoing surgery, 5 years survival and 10 years survival is reported respectively over 90 % and 67 %.

However, most patients experience a relapse [6]. In published studies the percentage of recurrence varies from 33 to 78 %, with lymph node metastases, distant metastases and a non-secretory carcinoma representing the most important negative prognostic factors.

Our case report is unusual for its presentation; in literature there are no cases of parathyroid carcinoma in patients with CRF (chronic renal failure) diagnosed by means of pulmonary metastasis.

The patient referred symptoms compatible with CRF; nodular lesions to parathyroid glands and an elevated PTH induced physician to hypothesize secondary hyperparathyroidism.

The presence of haemoptysis gave us suspicion for malignant neoplasm but only histological examination allowed us to recognize the nature of the lesion, because even PET-CT didn’t detect, an hypercaptation in the parathyroids.

## 4 Conclusions

The presented case is very characteristic for its clinical presentation.

In patients with CRF it’s difficult to diagnose parathyroid carcinoma, because its presentation mimics the most common secondary hyperparathyroidism; the concomitant presence of metastases should lead us to suspect malignant parathyroid lesions.

## Funding

None.

## Ethical approval

The study is exempt from ethnical approval.

## Consent

Written informed consent was obtained from the patient for publication of this case report and accompanying images.

## Author contribution

Nadia Innaro, Rita Gervasi and Lucia Stella Curto contributed to think up the design of manuscript; Francesca Caracciolo and Lucia Stella Curto contribute to literature research, data analysis and language revision. All authors wrote the manuscript.

## Registration of Research Studies

This case report doesn’t need registration.

## Guarantor

Lucia Stella Curto.

## Provenance and peer review

Not commissioned, externally peer-reviewed.

## Declaration of Competing Interest

None.
